# Retrospective, Multicenter Analysis Comparing Conventional with Oncoplastic Breast Conserving Surgery: Oncological and Surgical Outcomes in Women with High-Risk Breast Cancer from the OPBC-01/iTOP2 Study

**DOI:** 10.1245/s10434-021-10809-1

**Published:** 2021-10-13

**Authors:** Florian Fitzal, Michael Bolliger, Daniela Dunkler, Angelika Geroldinger, Luca Gambone, Jörg Heil, Fabian Riedel, Jana de Boniface, Camilla Andre, Zoltan Matrai, Dávid Pukancsik, Regis R. Paulinelli, Valerijus Ostapenko, Arvydas Burneckis, Andrej Ostapenko, Edvin Ostapenko, Francesco Meani, Yves Harder, Marta Bonollo, Andrea S. M. Alberti, Christoph Tausch, Bärbel Papassotiropoulos, Ruth Helfgott, Dietmar Heck, Hans-Jörg Fehrer, Markus Acko, Peter Schrenk, Elisabeth K. Trapp, Pristauz-Telsnigg Gunda, Paliczek Clara, Giacomo Montagna, Mathilde Ritter, Jens-Uwe Blohmer, Sander Steffen, Laszlo Romics, Elizabeth Morrow, Katharina Lorenz, Mathias Fehr, Walter Paul Weber

**Affiliations:** 1 Department of General Surgery and Breast Health Center, Medical University Vienna, Vienna, Austria; 2 Center for Medical Statistics, Informatics and Intelligent Systems, Medical University of Vienna, Vienna, Austria; 3 Departement of Obstetrics and Gynecology, Breast Center, Heidelberg University Hospital, Heidelberg, Germany; 4 Department of Molecular Medicine and Surgery, Karolinska Institutet, Stockholm, Sweden; 5 Departemt of Surgery, Capio St Göran’s Hospital, Stockholm, Sweden; 6 Department of Surgery, Uppsala University Hospital, Uppsala, Sweden; 7 Department of Breast and Sarcoma Surgery, National Institute of Oncology, Budapest, Hungary; 8 Federal University of Goiás, Araújo Jorge Hospital, Goiás Anti-Cancer Association, Goiás, Brazil; 9 National Cancer Institute Vilnius Lithuania, Vilnius, Lithuania; 10 Faculty of Medicine, University of Vilnius, Vilnius, Lithuania; 11 Centro di Senologia della Svizzera Italiana (CSSI), Lugano, Switzerland; 12 Department of Obstetrics and Gynecology, Ente Ospedaliero Cantonale (EOC), Lugano, Switzerland; 13 Department of Plastic, Reconstructive and Aesthetic Surgery, Ente Ospedaliero Cantonale (EOC), Lugano, Switzerland; 14 Faculty of Biomedical Sciences, Università della Svizzera Italiana (USI), Lugano, Switzerland; 15 Breast-Center, Zurich, Switzerland; 16 Department of Surgery, Ordensklinikum Linz – Sisters of Charity, Linz, Austria; 17 Department of Obstetrics and Gynecology, Medical University of Graz, Graz, Austria; 18 Breast Center, University Hospital of Basel, Basel, Switzerland; 19 Breast Service, Department of Surgery, Memorial Sloan Kettering Cancer Center, New York, NY USA; 20 Department of Gynecology and Breast Center, Charité University Hospital Campus Charité-Mitte, Berlin, Germany; 21 Clinical Cancer Registry, Charité Comprehensive Cancer Center (CCCC), University Medical Center Berlin, Campus Charité Mitte, Berlin, Germany; 22 New Victoria Hospital, NHS Greater Glasgow and Clyde, Glasgow, United Kingdom; 23 Department of Academic Surgery, University of Glasgow, Glasgow, United Kingdom; 24 Department of Gynecology and Obstetrics, Cantonal Hospital Frauenfeld, Frauenfeld, Switzerland; 25 University of Basel, Basel, Switzerland

## Abstract

**Introduction:**

Recent data suggest that margins ≥2 mm after breast-conserving surgery may improve local control in invasive breast cancer (BC). By allowing large resection volumes, oncoplastic breast-conserving surgery (OBCII; Clough level II/Tübingen 5-6) may achieve better local control than conventional breast conserving surgery (BCS; Tübingen 1-2) or oncoplastic breast conservation with low resection volumes (OBCI; Clough level I/Tübingen 3-4).

**Methods:**

Data from consecutive high-risk BC patients treated in 15 centers from the Oncoplastic Breast Consortium (OPBC) network, between January 2010 and December 2013, were retrospectively reviewed.

**Results:**

A total of 3,177 women were included, 30% of whom were treated with OBC (OBCI n = 663; OBCII *n* = 297). The BCS/OBCI group had significantly smaller tumors and smaller resection margins compared with OBCII (pT1: 50% vs. 37%, *p* = 0.002; proportion with margin <1 mm: 17% vs. 6%, *p* < 0.001). There were significantly more re-excisions due to R1 (“ink on tumor”) in the BCS/OBCI compared with the OBCII group (11% vs. 7%, *p* = 0.049). Univariate and multivariable regression analysis adjusted for tumor biology, tumor size, radiotherapy, and systemic treatment demonstrated no differences in local, regional, or distant recurrence-free or overall survival between the two groups.

**Conclusions:**

Large resection volumes in oncoplastic surgery increases the distance from cancer cells to the margin of the specimen and reduces reexcision rates significantly. With OBCII larger tumors are resected with similar local, regional and distant recurrence-free as well as overall survival rates as BCS/OBCI.

**Supplementary information:**

The online version contains supplementary material available at 10.1245/s10434-021-10809-1.

## Key Points

- *Question* Does oncoplastic breast conservation (OBC) with large resection volumes (OBCII; Clough level II/Tübingen 5-6) achieve better local recurrence rates than conventional breast conserving surgery (BCS) or low volume oncoplastic procedures (OBCI; Clough level I/Tübingen 3-4)?
- *Findings* Of 3,177 women, 30% were treated with OBC. The BCS/OBCI group had significantly smaller resection margins and higher re-excision rates. At a median follow-up of 74.5 months, however, there were no differences in recurrence or survival rates between the two groups.
- *Meaning* OBCII allows for resection of larger tumors without increasing local recurrence risk and reduces the re-excision rate. Margins larger than “no ink on tumor” do not improve local control.

Immediate techniques of oncoplastic surgery (iTOP) include immediate breast reconstruction after nipple/skin-sparing mastectomy (IBR) and oncoplastic breast conservation (OBC), including parenchymal rearrangement or volume replacement by adjacent perforator flaps designed to repair defects after breast-conserving surgery.^1,2^ OBC procedures may be divided into small (Clough level I, Tübingen 3,4) or extended (Clough level II, Tübingen 5,6) resections with removal of less or more than 20% of the breast tissue.^3–7^ Compared with conventional breast-conserving surgery (BCS), OBC allows resection of larger tumors and achievement of better cosmesis without delaying adjuvant therapies.^8–11^ Additionally, retrospective studies have shown that, compared with BCS, OBC significantly reduces the rate of positive margins resulting in lower reoperation rates.^9,12^ A recent, large, population-based study has shown that the use of OBC reduces the number of mastectomies.^13^

Currently international guidelines recommend “no ink on tumor” as a safe resection margin to achieve optimal local control (i.e., recurrence rate below 1%/year).^14–16^ These recommendations are based on a large meta-analysis that demonstrated higher local recurrence rates in patients with tumors touching the inked margin.^17,18^ However, the question about the optimal margin width after breast-conserving surgery remains open. Vicini et al.’s meta-analysis, including more than 55,000 women showed that a resection margin ≥2 mm was associated with a 56% reduction in ipsilateral breast cancer recurrence, similar as for DCIS, suggesting that larger margins may further reduce the risk of local relapse.^19–21^ We therefore hypothesized that extended OBC resections (Clough level II or Tübingen level 5 and 6) may improve local recurrence rates, in high-risk tumors, by increasing resection free margins (≥2 mm) compared with BCS and OBC level I.^3,4^ Because this hypothesis cannot be tested in a randomized controlled trial, the Oncoplastic Breast Consortium (OPBC) gathered to address this question using data collected within its members’ network of international breast cancer centers.^22^

## Materials and Methods

### Design

We performed a retrospective review of prospectively registered consecutive patients treated at 15 institutions of members of the OPBC network between January 2010 and December 2013. In case of missing data, patient charts were reviewed individually. Cases with ≥1 exclusion criteria, those with no definitive tumor biology, and those lost to follow-up were excluded (*n* = 197). The trial was first approved by the local ethic authorities from the Medical University Vienna (1468/2018) and thereafter by all local ethic authorities relevant to participating centers. Case report form (CRF)-related data were anonymized by the local sites and sent to the Medical University Vienna. After data cleaning by the principal investigator (F.F.), data were analyzed by three statisticians (W.H., A.G., and D.D.).

### Inclusion Criteria

1. Women aged ≥18 years, who had surgery between January 1, 2010 and December 31, 2013 with regular documented follow-up visits at least once a year
2. Histologically verified primary unilateral breast cancer
3. High-risk invasive cancer defined as having at least one of the following criteria:
  - Human epidermal growth factor receptor 2 (HER2)-positive or triple-negative (immunohistochemistry)
  - Genomic high-risk (PAM50, Endopredict, Mammaprint or Oncotype DX)
  - If endocrine-positive and HER2-negative, Ki67 ≥30% or high tumor grade
  - Lymph node-positive disease of any tumor biology
4. High-risk *in situ* cancer defined as high grade (DCIS G3, DIN III)
5. Having received breast-conserving surgery, reexcision due to unclear margins (R1/Rx) was allowed at any time

### Exclusion Criteria

1. Stage IV breast cancer
2. Omission of adjuvant breast radiotherapy when recommended
3. Local recurrence defined as an in-breast recurrence within 5 years from surgery for a primary breast cancer
4. Pathogenic BRCA mutation (if genetic testing was available)
5. Positive margins defined as “ink on tumor” (R1) without reexcision
6. Mastectomy

### Surgical Groups

Two different surgical groups were created according to the Hoffmann Wallwiener Tübingen classification,^4^ as recommended by the OPBC^6^:

BCS/OBCI—conventional breast conservation Tübingen 1 and 2 (no oncoplastic surgery), and low-volume oncoplastic breast conservation Tübingen 3 and 4 (<20% resection volume). These two groups had similar clinicopathological features and 5-year local recurrence rates (Supplementary Table S1 and Fig. S1).

OBCII—high-volume oncoplastic breast conservation Tübingen 5 and 6 (>20% resection volume).

Because Tübingen 3 and 4 usually are low-volume level I Clough resections, such as batwing, doughnut, or local intraparenchymal flaps without extensive resections, we classified all of Tübingen 1-4 and the conventional breast conservation group together as BCS/OBCI. Tübingen level 5 and 6 or Clough level II are usually oncoplastic resections combined with extensive breast reduction mammaplasties, such as the inverse-T Eren technique or Hall Findlay technique^23,24^ and were classified as high-volume oncoplastic breast conservation (OBCII).

### Oncologic Endpoints

1. Local breast cancer recurrence rate (LBCR) including ipsilateral in-breast cancer events, invasive and noninvasive
2. Regional breast cancer recurrence rate (RBCR) defined as regional lymph node recurrence within the ipsilateral axilla
3. Distant disease-free survival (DDFS), including distant invasive breast cancer events
4. Overall survival (OS) (death from any cause)

### Perioperative Endpoint

1. Number of reexcisions due to positive or unclear margins (R1/Rx; women with a pathologic complete response were included into the Rx group)
2. Tumor-free resection margin width in mm, comparing <1 mm, 1–3 mm, and >3 mm

### Statistical Analysis

Categorical variables are presented as counts and percentages and continuous ones as medians with first and third quartiles. Depending on the scale and distribution, the Chi-square test, *t*-test, or Mann-Whitney test were applied to compare BCS/OBC I with OBC II. Kaplan-Meier curves were used to visualize survival proportions after surgery. Median follow-up time was estimated by reversing the roles of deaths and censoring. Cox regression was applied to model the effect of type of surgery [BCS/OBC I vs. OBC II] on survival. The proportional hazards assumption was evaluated using plots of scaled Schoenfeld residuals versus rank of time. Because of nonproportional hazards, weighted Cox regression was applied, which estimates an average hazard ratio.^25^ For oncological outcomes, the competing risk of death was considered in the statistical models. Cumulative incidence functions were used to depict the three oncologic outcomes in the two types of surgery. Hypotheses of equality of cause-specific cumulative incidence functions between the two groups are evaluated with Gray’s test. The Fine & Gray model was applied to estimate the subdistribution hazard, and additionally, the cause-specific (death-censored) Cox regression model was estimated.

Following the recommendation of Wolbers et al.^26^, for the purposes of prognosis and medical decision-making, the subdistribution hazard is of primary interest, because it quantifies the absolute risks of the event of interest. The cause-specific hazard directly models the effect of the covariate on event rates among people at risk and is of interest for etiological research questions. All statistical models were adjusted by the following known risk factors and potential confounders: age, tumor biology, tumor size, nodal status, invasive versus noninvasive cancer, and systemic treatment. For LBCR, additional models were estimated with the added confounders margin width and reexcision due to R1. A robust sandwich covariance matrix estimate was used to account for the intracluster dependence of this multicenter study. Two-sided *p*-values <0.05 were considered statistically significant. SAS 9.4 and R 4.0.2 was used for statistical analysis.

## Results

### Participating Breast Centers

We included 3,177 patients from 15 different institutions in 8 different countries (Austria *n* = 824, Brazil *n* = 54, Germany *n* = 728, Hungary *n* = 50, Lithuania *n* = 284, Sweden *n* = 313, Switzerland *n* = 682, United Kingdom *n* = 242). Thirty percent of patients were treated with OBC, 297 (9.3%) of whom with OBC II and 663 (20.9%) with OBC I, whereas 2217 (69.8%) received BCS. Four institutions included 75% of all OBCII, five institutions had less than 5% of OBCII cases, and four centers had none.

### Clinicopathological Characteristics

The great majority of all patients (92.3%) had invasive cancer while the remaining had high-grade DCIS. Twenty seven percent were aged ≤50 years and 19% were >70 years. Sixteen percent of patients had received neoadjuvant chemotherapy. Tumor size was ≥2 cm in 40% of cases. Node positivity was confirmed on final pathology in 50% of cases. A minority of tumors (6.6%) were invasive lobular breast cancer and Luminal A (9%; all of these patients were nodal positive), whereas 41% were Luminal B, 27% were HER2+, and 21% were triple-negative. Compared with the BCS/OBCI group, patients treated with OBCII were more likely to have larger tumors and to be node-positive. Tumor size before neoadjuvant therapy was, however, similar in both groups. Tumor biology also differed among surgical groups: HER2+ tumors were more frequent in the OBCII group; triple-negative tumors were more common in the BCS/OBCI group (Tables 1, 2).

**Table 1:** Clinicopathological features

	BCS/OBCI		OBCII		*p* value
	*n*	***%***	*n*	***%***	
Entire cohort	2880	**91**	297	**9**	
Age (yr)	2879	**58 [49-68]**	297	**54 [46-63]**	**<0.001**
Invasive cancer	2673	**93**	260	**88**	**0.0012**
Lobular histology	189	**7**	21	**7**	0.7372
NAC	441	**16**	65	**25**	**0.0032**
Tumor size (mm)^*^	379	**30 **[23-40]	41	**30 [23-43]**	0.4515
cT1/2^*^	363	**86**	34	**77**	0.1099
Radiotherapy	2652	**92**	293	**98**	**<0.001**
Radiotherapy boost	2033	**70**	197	**66**	0.1264
Endocrine therapy	1927	**67**	196	**66**	0.7494
Chemotherapy	1752	**61**	186	**63**	0.5464

Categorical variables are presented as counts (%) and continuous ones as medians (IQR)

Statistically significant values are indicated in bold

*Refers to patients treated with neoadjuvant chemotherapy only

*BCS* conventional breast-conserving surgery (Tübingen 1-2); *OBCI* oncoplastic breast-conserving surgery level I (Clough level I/Tübingen 3-4); *OBCII* oncoplastic breast-conserving surgery (Clough level II/Tübingen 5-6); *NAC* neoadjuvant chemotherapy

**Table 2:** Clinicopathological features

	BCS/OBCI		OBCII		*p* value
	*n*	*%*	*n*	*%*	
Entire cohort	2880	**91**	297	**9**	
Pathological T stage					
pTis	231	**8**	34	**12**	**<0.001**
pT1	1367	**50**	103	**38**	
pT2	1014	**37**	119	**43**	
pT3/4	106	**4**	18	**7**	
Pathological N stage					
pN0	1357	**49**	103	**37**	**<0.001**
pN1	1173	**42**	163	**58**	
pN2/3	241	**9**	16	**6**	
Subtype					
Luminal A	272	**10**	18	**6**	**<0.001**
Luminal B	1209	**43**	104	**36**	
Luminal HER2+	505	**18**	80	**27**	
non-luminal HER2+	232	**8**	40	**14**	
Triple negative	611	**22**	50	**17**	

Categorical variables are presented as counts (%). Statistically significant values are indicated in bold

### Surgical Outcomes

**Fig. 1:**
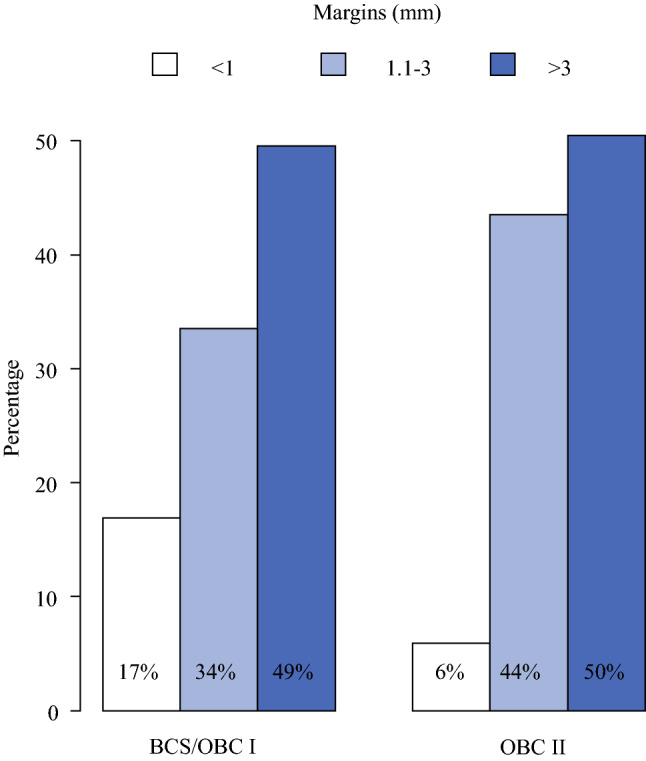
Margin status by type of surgery. Result from Chi-square test; 15% (*n* = 464) of data are missing. *BCS* conventional breast-conserving surgery (Tübingen 1-2); *OBCI* oncoplastic breast-conserving surgery level I (Clough level I/Tübingen 3-4); *OBCII* oncoplastic breast-conserving surgery (Clough level II/Tübingen 5-6)

**Fig. 2:**
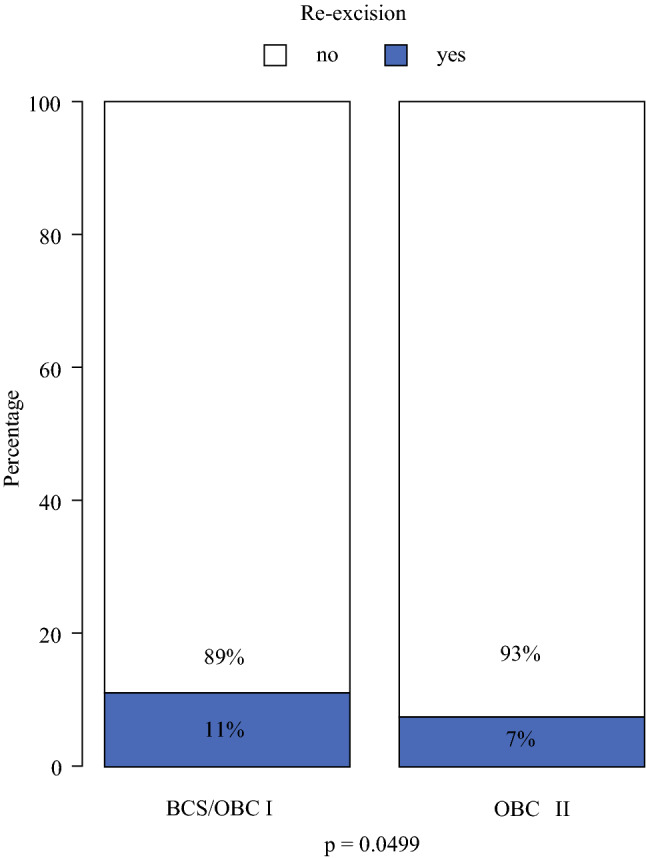
Reexcision rates by type of surgery. Result from Chi-square test; no data are missing. *BCS* conventional breast-conserving surgery (Tübingen 1-2); *OBCI* oncoplastic breast conserving surgery level I (Clough level I/Tübingen 3-4); *OBCII* oncoplastic breast conserving surgery (Clough level II/Tübingen 5-6)

Margin width differed between the two groups: 17% had a margin <1 mm in the BCS/OBCI group versus 6% in OBCII group (*p* = 0.001; Fig. 1) as did the number of reexcisions due to positive margins (tumor on ink) after the first surgical attempt (11% in BCS/OBCI vs. 7% in OBCII, *p* = 0.025; Fig. 2).

### Oncological Outcomes

**Fig. 3:**
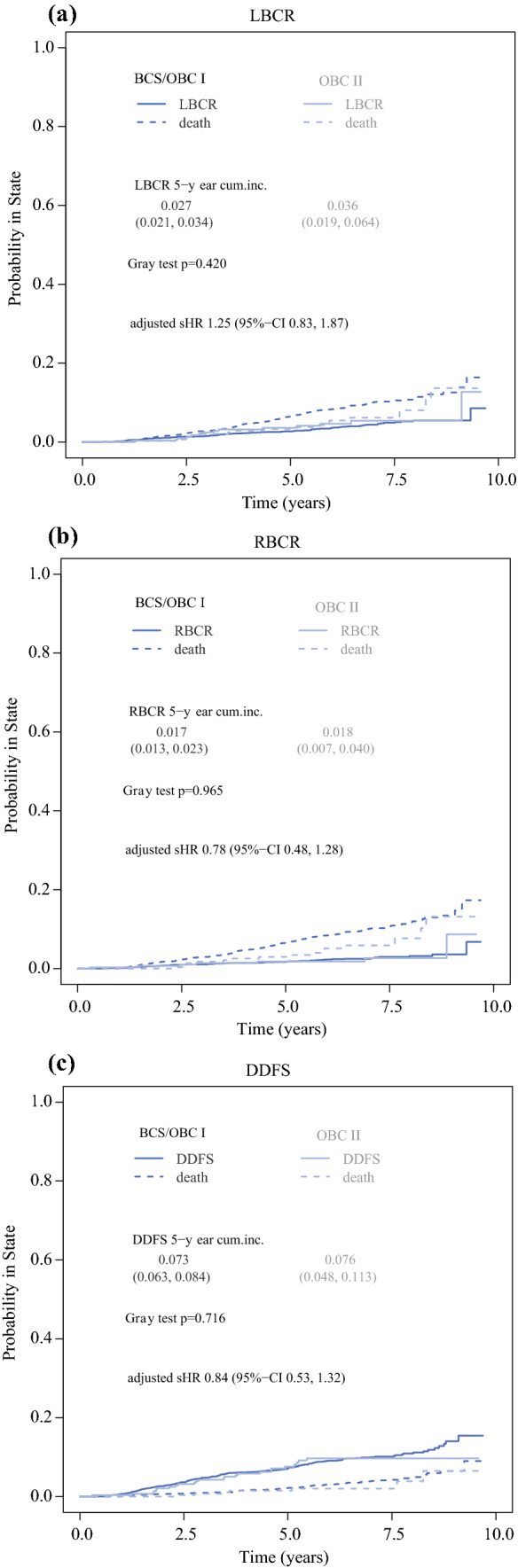
**a** Cumulative incidence plot of LBCR by type of surgery. **b** Cumulative incidence plot of RBCR by type of surgery. **c** Cumulative incidence plot of DDFS by type of surgery. *BCS* conventional breast-conserving surgery (Tübingen 1-2); *OBCI* oncoplastic breast-conserving surgery level I (Clough level I/Tübingen 3-4); *OBCII* oncoplastic breast-conserving surgery (Clough level II/Tübingen 5-6); *LBCR* Local breast cancer recurrence rate; *RBCR* regional breast cancer recurrence rate; *DDFS* distant disease-free survival

The median follow-up time was 74.5 months (interquartile range [IQR] 60.32–89.56). During follow-up, 3.8% of patients (*n* = 119) developed local recurrence, 2.3% (*n* = 72) developed regional recurrence, and 8.8% (*n* = 253) developed distant recurrence. Two hundred seventy-eight (8.8%) patients died during the study period. Unadjusted cumulative incidence rates showed no significant differences in all oncologic outcomes for OBCII versus BCS/OBCI (Figs. 3a-c). The 5-year LBCR was 2.7% (2.1–3.4%) in the BCS/OBCI group and 3.6% (1.9–6.4%) in the OBCII group (*p* = 0.420). The 5-year distant recurrence rates were 7.3% (6.3–8.4%) in the BCS/OBCI group and 7.6% (4.8–11.3%) in the OBCII group (*p* = 0.716), whereas 5-year regional recurrence rates was 1.7% (1.3–2.3%) and 1.8% (0.7–4.0%; *p* = 0.965). Multivariable time-to-event analyses (Fine & Gray model and cause-specific Cox regression) for RBCR, DDFS, and LBCR showed that OBCII was not independently associated with any of these endpoints (Table 3).

**Table 3:** Multivariable associations for oncological outcomes

	n (%)^*^	Cause-specific hazard		Subdistribution hazard	
		Univariate model	Multivariable model^**^	Univariate model	Multivariable model^**^
LBCR	2834 (97)	1.23 (0.67, 2.07), *p* = 0.470	1.33 (0.67, 2.39), *p* = 0.380	1.26 (0.89, 1.78), *p* = 0.194	1.40 (0.96, 2.05), *p* = 0.080
RBCR	3175 (72)	0.989 (0.41, 2.01), *p* = 0.978	0.731 (0.27, 1.59), *p* = 0.473	(0.62, 1.66), *p* = 0.473	0.78 (0.48, 1.27), *p* = 0.33
DDFS	2872 (253)	1.00 (0.58, 1.37), *p* = 0.699	0.84 (0.52, 1.31), *p* = 0.441	0.92 (0.61, 1.39), *p* = 0.710	0.84 (0.56, 1.27), *p* = 0.429
LBCR^***^	2561 (78)		1.19 (0.53, 2.66), *p* = 0.677^***^		1.25 (0.83, 1.87), *p* = 0.292^***^

All models were corrected for the intracluster correlation of breast center

*Sample size of multivariable model

**Adjusted for subtype, pT, pN, invasive vs. noninvasive, and neoadjuvant therapy vs. adjuvant

***Additionally, adjusted for margin width (mm) and reoperation due to R1

*LBCR* local breast cancer recurrence rate; *RBCR* regional breast cancer recurrence rate; *DDFS* disease-free survival

## Discussion

This retrospective, multicenter analysis of 3,177 high-risk breast cancer patients treated in 15 different institutions demonstrated no significant differences in local recurrence-free survival comparing large-volume oncoplastic surgery with conventional breast-conserving surgery or low-volume oncoplastic surgery. Large-volume oncoplastic surgery, however, increased the tumor-free margin width and significantly reduced the number of reexcisions due to R1 at the first surgical attempt.

The use of OBC has increased over time.^27^ In 2014, 33% of all breast-conserving surgeries were performed using any type of oncoplastic surgery.^28^ Therefore, breast surgeons in training are increasingly exposed to these techniques, e.g., in the United Kingdom all newly appointed consultant breast surgeons have to be familiar with mammaplasty techniques.^29^ There are three main reasons for this. First, the resection of tumors in the medial as well as central and caudal regions of the breast may lead to nipple as well as breast distortion. OBC helps to restore the natural breast shape and to fill larger defects, improving perception of one’s breast and cosmetic outcome.^23^ Second, the concomitant breast reduction using OBC in very large breasts may reduce radiotherapy side effects common in this subgroup as reported in prospective trials.^30^ Finally, OBC allows the resection of larger tumors without compromising oncologic outcome,^9,11^ as confirmed in this study, and may thus reduce the necessity of mastectomy in selected patients. In fact, two large retrospective analyses were able to demonstrate for the first time that there seems to be a trend toward lower mastectomy rates with increasing OBC rates.^13,31^

However, prospective data demonstrating a significant improvement in patient-reported outcome measurements (PROMs) after oncoplastic surgery are still lacking.^32–34^ A meta-analysis published in 2013 revealed that most oncoplastic studies are nonrandomized, uncontrolled analyses with poor design, lack of robust data, and insufficient statistical power.^35^ There is only indirect evidence that OBC may improve PROMs. Several authors reported an association between breast symmetry, breast shape, and cosmesis with anxiety, depression, body image, sexuality, and self-esteem.^36,37^ Removal of large tumors by mastectomy is associated with dissatisfaction with breast cosmesis, which further correlated with an increased depression score in Asian women.^38^ Breast symmetry itself significantly correlates with depression scores,^39^ suggesting that improving breast cosmesis combined with contralateral symmetrization, as commonly done in OBC, may improve breast self-esteem and depression scores.

The observation that oncoplastic surgery increases tumor-free margin width and reduces reoperation rates in our analysis is in line with several other retrospective data.^9,12,29^ This is especially true when using OBCII in breasts with cup size C or larger. Larger resections, however, are accompanied by a significantly increased risk, of postoperative morbidity, namely up to 30%.^1^ In a prospective, nonrandomized, controlled trial (iTOP1), we were able to demonstrate that OBC, performed for large breast tumors, results in similar breast self-esteem scores and similar quality of life compared with BCS,^33^ demonstrating that an increased morbidity rate does not influence long-term quality of life. However, morbidity depends on the extent of oncoplastic surgery, with higher morbidity rates in OBCII, according to Clough classification.^3^ Other authors have not reported significant increases in clinically relevant morbidity in large retrospective analyses.^31^

Our study failed to demonstrate an association of large oncoplastic resections in high-risk invasive breast cancer patients with better LBCR due to increased margin width. This is in line with the large retrospective analysis by Houssami et al.,^17,18^ showing no association of margin width with local control. In this respect the definition of “no ink on tumor” for an R0 resection remains true^14^ even in larger and high-risk tumors.

There is an ongoing debate and limited data regarding optimal resection margins after neoadjuvant chemotherapy.^16,40–42^ In our study, we found no significant difference regarding local recurrence in women with and without neoadjuvant chemotherapy (4.9% vs. 3.4%; *n* = 1,920). OBCII, despite larger resection margins, was not associated with a lower local recurrence rate after neoadjuvant therapy. Thus, our data support the current evidence that “no ink on tumor” is an appropriate margin width also in patients receiving neoadjuvant chemotherapy.^43–46^

Landmark trials investigating the safety of breast-conserving surgery only included smaller tumors (up to 2 cm in size),^47,48^ whereas larger tumors were less frequently studied.^49–51^ Moreover, tumor biology was unknown in these trials; thus, there is a lack of robust data supporting the use of breast-conserving surgery for high-risk pT2/3 tumors. In our study of 3,177 women, 35% of patients had a tumor larger than 2 cm on final pathology, and the great majority had an aggressive subtype (Luminal B, HER2+ or triple-negative tumors). The small percentage of luminal A tumors included in our study were node-positive. The overall local recurrence rate, at 74 months of follow-up, after breast-conserving surgery and radiotherapy was 3.6%. The regional recurrence rate was 2.2%, and 9.5% developed a distant relapse and 9.3% deceased during the study period.

Our results are in line with those reported by Andre and colleagues who compared local recurrences and survival rates between patients undergoing simple and complex OBC versus conventional BCS and found no differences among the three groups.^52^

Limitations of this study include the retrospective nature and the low number of local in-breast events (*n* = 121 compared with the expected *n* = 230). Additionally, differences in demographic data between the surgical groups limited the analyses of oncological outcomes. The statistical testing for superiority gives no answer to the question of noninferiority for one of the two groups. However, we believe that even with an increased number of events and included participants our results would not change. Another limitation of our study is that we did not evaluate race distribution; however, in Central Europe, race heterogeneity is much smaller compared with the United States and racial disparities therefore are understudied. Future studies from the OPBC will assess possible race disparities in oncoplastic surgery.

Strengths of the study are the large sample size, the multicentric, international, design, and the adjustment for several important oncologic variables. The included patients were treated in eight different countries outside clinical trials, making our results highly generalizable to the real-world scenario.

## Conclusions

Our study shows that oncoplastic level II resections in high-risk breast cancer patients increase margin width but is not associated with lower local recurrence rates. The number of reexcisions due to R1 however was significantly lower in oncoplastic level II techniques. Our data support the use of breast-conserving surgery techniques for women with tumors ≥2 cm irrespectively of tumor biology and receipt of neoadjuvant chemotherapy as long as “no ink on tumor” margins are obtained to achieve optimal local and distant control.

## Supplementary information

Supplementary information

## Declarations

### Data Access, Responsibility, and Analysis

F. Fitzal had full access to all the data in the study and takes responsibility for the integrity of the data and the accuracy of the data analysis.

### Prior Presentation

This study was presented in poster format at the 17^th^ St. Gallen International Breast Cancer Conference 2021 (virtual meeting) as well as at the annual congress of the Austrian Society of Surgery 2021, virtual meeting.
