# Supplementary material for: Retrospective, Multicenter Analysis Comparing Conventional with Oncoplastic Breast Conserving Surgery: Oncological and Surgical Outcomes in Women with High-Risk Breast Cancer from the OPBC-01/iTOP2 Study
Source: Ann Surg Oncol. 2021 Oct 13;29(2):1061–70. doi: 10.1245/s10434-021-10809-1 (PMC8724061; doi:10.1245/s10434-021-10809-1)
Supplement: Supplementary file 1 — Supplementary information [file 10434_2021_10809_MOESM1_ESM.docx]

**SUPPLEMENTARY DATA**

S**upplementary Table S1a.** **Clinicopathological features of the BCS vs OBCI group**

Categorical variables are presented as counts (%) and continuous ones as medians (IQR)

^*^Refers to patients treated with neoadjuvant chemotherapy only

BCS, conventional breast conserving surgery (Tübingen 1-2); OBCI, oncoplastic breast conserving surgery level I (Clough level I/Tübingen 3-4);

|  | **BCS**  **(n=2217)** | | **OBCI**  **(n=663)** | |
| --- | --- | --- | --- | --- |
|  | n | % | n | % |
| **Age** | 2216 | 59 [49-68] | 663 | 56 [48-66] |
| **Lobular Histology** | 139 | 6 | 50 | 8 |
| **Tumor size (mm)^*^** | 271 | 30 [22.5-35] | 108 | 30.5 [24.8-49.2] |
| **cT1/2^*^** | 270 | 88 | 93 | 81 |
| **Radiotherapy** | 2014 | 91 | 638 | 96 |
| **Endocrine**  **Therapy** | 1468 | 66 | 459 | 69 |
| **Chemotherapy** | 1349 | 61 | 403 | 61 |

S**upplementary Table S1b.** **Clinicopathological features of the BCS vs OBCI group**

Categorical variables are presented as counts (%)

BCS, conventional breast conserving surgery (Tübingen 1-2); OBCI, oncoplastic breast conserving surgery level I (Clough level I/Tübingen 3-4);

|  | **BCS**  **(n=2217)** | | **OBCI**  **(n=663)** | |
| --- | --- | --- | --- | --- |
|  | *n* | ***%*** | *n* | ***%*** |
| **Pathological T stage** |  |  |  |  |
| **pTis** | 161 | 8 | 70 | 11 |
| **pT1** | 1086 | 52 | 281 | 45 |
| **pT2** | 772 | 37 | 242 | 39 |
| **pT3/4** | 78 | 4 | 28 | 5 |
| **Pathological N stage** |  |  |  |  |
| **pN0** | 1016 | 48 | 341 | 54 |
| **pN1** | 945 | 44 | 228 | 36 |
| **pN2/3** | 177 | 8 | 64 | 10 |
| **Subtype** |  |  |  |  |
| **Luminal A** | 211 | 10 | 61 | 9 |
| **Luminal B** | 933 | 43 | 276 | 42 |
| **Luminal HER2+** | 361 | 17 | 144 | 22 |
| **Non Luminal HER2+** | 182 | 8 | 50 | 8 |
| **Triple negative** | 489 | 22 | 122 | 19 |

**Supplementary Figure S1. Cumulative incidence plot of LBCR by type of surgery (BCS vs OBCI group.**

BCS, conventional breast conserving surgery (Tübingen 1-2); OBCI, oncoplastic breast conserving surgery level I (Clough level I/Tübingen 3-4); LBCR, Local breast cancer recurrence rate
